# Combining label-free Raman spectroscopy and machine learning to identify early biomarkers of COVID-19 disease severity and mortality

**DOI:** 10.1117/1.JBO.31.4.046005

**Published:** 2026-04-15

**Authors:** Maryam Heidarifard, Katherine Ember, Frédérick Dallaire, Elsa Brunet-Ratnasingham, Yiheng Chen, Nassim Ksantini, Myriam Mahfoud, Guillaume Sheehy, Hugo Soudeyns, Philippe Jouvet, Sze Man Tse, Caroline Quach, Brent Richards, Daniel E. Kaufmann, Frédéric Leblond, Mathieu Dehaes

**Affiliations:** a Centre de Recherche Azrieli du CHU Sainte-Justine, Montreal, Quebec, Canada; b Université de Montréal, Institute of Biomedical Engineering, Montreal, Quebec, Canada; c CHU Montreal, Research Centre, Montreal, Quebec, Canada; d Polytechnique Montréal, Department of Engineering Physics, Montreal, Quebec, Canada; e Université de Montréal, Department of Microbiology, Infectiology and Immunology, Montreal, Quebec, Canada; f McGill University, Departments of Human Genetics, Epidemiology, and Biostatistics, Montreal, Quebec, Canada; g Jewish General Hospital, McGill University, Lady Davis Institute, Montreal, Quebec, Canada; h 5 Prime Sciences, Montreal, Québec, Canada; i Emory University School of Medicine, Department of Pathology and Laboratory Medicine, Pathology Advanced Translational Research Unit, Atlanta, Georgia, United States; j Université de Montréal, Department of Pediatrics, Montreal, Quebec, Canada; k Université de Montréal, Department of Microbiology, Infectious Diseases and Immunology, Montreal, Quebec, Canada; l McGill University, Department of Medicine, Montreal, Quebec, Canada; m King’s College London, Department of Twin Research, London, United Kingdom; n Lausanne University Hospital and University of Lausanne, Division of Infectious Diseases, Lausanne, Switzerland; o Université de Montréal, Department of Medicine, Montreal, Quebec, Canada; p Université de Montréal, Department of Radiology, Radio-oncology and Nuclear Medicine, Montreal, Quebec, Canada

**Keywords:** COVID-19, Raman spectroscopy, machine learning modeling, plasma, biomarkers, disease mortality, disease severity

## Abstract

**Significance:**

Early prediction of COVID-19 severity and mortality is crucial for optimizing clinical care and patient outcomes, but remains challenging.

**Aim:**

We aim to develop a screening tool combining label-free Raman spectroscopy and machine learning modeling to predict COVID-19 severity and mortality.

**Approach:**

Patients infected by SARS-CoV-2 (N=58) were recruited during the first wave of COVID-19 and stratified based on respiratory support. Blood samples were collected during hospitalization and analyzed using Raman spectroscopy and metabolomics. Machine learning models based on Raman spectra were developed to classify (1) survivors versus nonsurvivors, (2) critical patients with noninvasive versus invasive ventilation, and (3) noncritical (no respiratory support or oxygen via nasal cannula) versus critical patients.

**Results:**

Raman peaks assigned to proteins, glucose, lactic acid, fatty acids, urea, and lipids were extracted by the models. Area under the receiver operating characteristic curve ranged between 0.83 and 0.94, with sensitivities and specificities ranging between 80% and 83% and 75% and 92%, respectively. Accuracy for detecting mortality, invasive ventilation, and critical disease was 90%, 87%, and 78%. A complementary metabolomic analysis confirmed some molecular differences between groups.

**Conclusions:**

These results suggest the potential of Raman spectroscopy and machine learning modeling to stratify COVID-19 patients at admission, individualize care, and improve survival rates.

## 1 Introduction

The COVID-19 pandemic was caused by the severe acute respiratory syndrome coronavirus 2 (SARS-CoV-2) and posed a significant threat to global health. As of February 2026, the number of confirmed COVID-19 cases worldwide had surpassed 779 million, resulting in more than 7 million confirmed deaths.^1^ Despite the efficacy of vaccines in reducing disease severity and mortality rates, controlling the spread of COVID-19 remains a challenge due to the virus’s ability to mutate.^2^ The emergence of viral mutations has led to varying infectivity patterns, further disrupting critical health services. Approximately 20% of COVID-19 patients experienced respiratory distress and required hospitalization, whereas the remainder exhibited mild symptoms.^3^ Hospitalized patients were at risk for multiorgan failure and death. To date, no consensus exists on identifying or predicting which critically ill patients are likely to recover and which ones are at a greater risk of mortality. This uncertainty is partly due to the complexity and heterogeneity of patient responses, as well as the dynamics of biological changes induced by the virus.^4^ Early prediction of disease severity and progression may allow for optimized patient management and potentially improve survival rates.

Pathogen-induced activation of the immune system increases energy demands and triggers the production of essential biomolecules, including amino acids, glucose, and fatty acids.^5^ Alterations in plasma amino acid levels may influence various metabolic pathways within the immune system, potentially affecting disease morbidity and mortality.^6^^–^^8^ Disturbances in other biomolecules, including DNA/RNA, lipids, and lipoproteins, were also observed in COVID-19 patients and can be detected in biofluids.^9^^–^^20^ The analysis of these dysregulated biomolecules could help to predict disease progression and develop novel therapeutic strategies to treat COVID-19 patients.

Raman spectroscopy (RS) is an emerging optical technique for detecting and identifying metabolites and biomolecules, including phospholipids, lipoproteins, DNA/RNA, and amino acids. It is a label-free and rapid screening tool that does not require biochemical reagents.^21^ The method is non-destructive and based on the excitation of molecules by light illumination and the determination of their molecular vibrational modes via the detection of scattered light.^22^^,^^23^ Previous studies have demonstrated the capability of RS to detect molecular changes associated with SARS-CoV-2 infection in non-hospitalized COVID-19 patients. These studies used saliva or serum to differentiate positive and negative COVID-19 patients.^15^^,^^24^^–^^28^ Confocal Raman spectroscopy was also applied to differentiate blood plasma between non‐severe and severe COVID-19 patients.^29^

Machine learning (ML) modeling may represent a useful tool for predicting clinical outcomes in COVID-19 patients. Previous studies have reported ML predictive models for various aspects of the disease, including diagnosis, severity, length of hospital stay, intensive care unit (ICU) admission, and mechanical ventilation outcomes.^30^^,^^31^ Imaging, comorbidity profiles, laboratory results, demographics, and clinical data were also previously used to predict patient outcomes.^32^^–^^38^ Machine learning has also been combined with Raman spectra to detect the biochemical perturbations due to the virus in the blood plasma, serum, and saliva.^15^^,^^28^^,^^39^^,^^40^ The accuracy of these models has ranged between 0.89 and 0.98. However, to our knowledge, RS and ML were not previously combined to predict COVID-19 mortality. Rapid and accurate prediction of COVID-19 severity and mortality could help in patient triaging and treatment and to better understand the molecular mechanisms underlying COVID-19-associated mortality.

The primary objective of this study was to develop a screening tool combining label-free RS and ML modeling (RS-ML) to predict COVID-19 severity and mortality. We hypothesized that this combined approach would provide an accurate and early prognostic of disease progression and clinical outcomes. To further interpret the RS-ML findings, biomolecular features extracted by the predictive models were compared with a metabolomics analysis of the same samples.

## 2 Material and Methods

### 2.1 Patients

Fifty-eight (N=58) patients who tested positive for SARS-CoV-2 with reverse transcription polymerase chain reaction (RT-PCR) and admitted to the *Centre hospitalier de l’Université de Montréal* (CHUM, Montreal, Canada) were enrolled in this study. Written informed consent was obtained from all patients by the institutional research ethics review board at the CHUM Research Centre (CRCHUM). Raman spectra acquisition and analyses were conducted at *Centre de recherche Azrieli du CHU Sainte-Justine* and approved by its research ethics review board. Patients were initially recruited as a prospective cohort study through the *Biobanque québécoise de la COVID-19 (BQC-19)*^41^ to investigate the clinical and biological factors affecting COVID-19 patients. Blood samples were collected at 11 (±5) days after symptom onset following clinical worsening. Patients were stratified based on the respiratory support required at the time of blood sampling. At this time, a clinical infectious disease physician evaluated the disease severity using a custom-developed disease severity scale. Noncritical disease status had SARS-CoV-2 infection that required hospitalization without supplemental oxygen (severity score 0) or had a mild-to-moderate disease that required a nasal cannula for oxygen (severity score 1). Critical disease status included hospitalized patients with non-invasive ventilation (severity score 2), invasive ventilation via endotracheal intubation (severity score 3), or extracorporeal membrane oxygenation (severity score 4). Mortality was assessed at day 60 after symptom onset (DSO60).

### 2.2 Sample Collection

Blood was collected in acid-citrate dextrose tubes and processed at CHUM (centrifuged at 850 g [2000 rpm] for 10 min at room temperature). Isolated plasma samples were used for Raman spectroscopy and metabolomics analysis. Plasma samples were biobanked and conserved at −80°C prior to utilization. Further analyses using these samples were blinded to patient clinical status, including mortality.

### 2.3 Raman Spectroscopy Data Acquisition and Analysis

The Raman system was based on light illumination at 785 nm, and scattered light was captured in the range of 600 to $1700\;{\mathrm{cm}}^{-1}$. Light was emitted via a laser (IPS, NJ, USA) powered at 100 mW, which allowed a high signal-to-noise ratio while avoiding sample deterioration. Light was collected via a spectrometer (Wasatch Photonics, NC, USA), including a CCD camera of 1024 pixels. Plasma samples were positioned at 11 mm from the laser tip in a 400  μL enhanced chemical-resistant aluminum cuvette that minimized contamination from inelastic scattering signals from the walls of the cuvette. Raman spectra were recorded by direct sample illumination. Experiments were performed to minimize plasma quantity (100  μL) and used a controlled thawing process: plasma samples were transferred from dry ice to regular ice for 30 min and rested at room temperature for an additional 30 min. Vortexing was limited to 1 min. Spectral acquisition parameters were optimized by averaging 20 measurements and adjusting the integration time between 300 and 1000 ms. Raman spectral data were acquired for 20 s or less. Liquid plasma samples were selected as they provided a high signal-to-noise ratio.

Standard preprocessing was applied to the spectra acquired for each plasma sample: (1) signal averaging, (2) cosmic ray removal, (3) baseline correction by background subtraction of the autofluorescence signal using the custom background removal algorithm *BubbleFill*,^42^ (4) smoothing using a Savitzky-Golay filter of order 3 with a window size of 11,^43^ and (5) standard normal variate normalization of the spectra.^44^ Codes were developed in Python 3.9.7 software.

### 2.4 Feature Selection and Machine Learning Classification

A linear support vector machine (SVM) with L1 regularization was used to reduce the number of features from 1100 to less than 20. Within this range, there were 26 peaks included in a universal library. These peaks were fitted with Gaussian functions such that the spectrum was reduced to the full width at half maximum of these 26 peaks. The weight of each universal peak was provided by the L1-regularized SVM. This approach allowed us to eliminate redundant features (i.e., Raman peaks consisting of multiple spectral bins) and those that did not provide useful information (in-between peaks region). Models were adjusted for age and sex as these variables were previously identified as risk factors for death or critical illness.^45^^,^^46^ A binary variable with a value equal to 1 for patients with at least one metabolic risk factor (diabetes type II, obesity, overweight, hypertension, or dyslipidemia) and 0 for patients with none of them was created and also used for multivariate modeling. Due to the lower prevalence of chronic diseases, only metabolic diseases were considered. A gradient boosting model was employed, and used Raman data, age, sex, and metabolic risk factors as inputs. It was selected for its efficiency in handling mixed data (spectral and demographic clinical information) and its ability to capture nonlinear relationships while minimizing overfitting through ensemble averaging and regularization.^47^ Hyperparameters were optimized through a grid search that ran over various combinations and included class weight, learning rate, number of estimators, minimum loss reduction, L1 and L2 regularization terms, and maximum tree depth. To account for comparisons involving imbalanced sample sizes $N_{1}$ and $N_{2}$ ($N_{1}>N_{2}$), the class weight hyperparameter was defined by the ratio $N_{1}/N_{2}$ for the class with $N_{1}$ patients, whereas the class weight equaled 1 for the class with $N_{2}$ patients. During data training, the penalty term associated with the misclassification of the class with $N_{1}$ samples was higher than that of a model, which involved balanced datasets (class weight ratio ∼1). To evaluate the performance of the models, a five-fold cross-validation was conducted. Sensitivity and specificity were extracted from the receiver operating characteristic (ROC) curve. Accuracy was calculated from the confusion matrix. The model with the parameters set that produced the highest area under the ROC curve (AUC) while optimizing for sensitivity and specificity was selected as the final model for each classification scenario. The AUC and its corresponding confusion matrix were reported with selected features.

### 2.5 Metabolomics Analysis

Metabolomics analysis was performed by a third party (Metabolon Inc, NC, USA) using ultraperformance liquid chromatography-mass spectroscopy (UPLC-MS, Metabolon HD4). Reference library included >1458 metabolites covering >70 metabolic pathways. Quality checks and curation of the metabolomics data were applied to ensure accurate and consistent identification of appropriate chemical entities and to remove those representing systemic artifacts, mis-assignments, and background noise.

### 2.6 Statistical Analysis

The nonparametric Mann-Whitney U test was used to compare clinical variables or Raman signals at peaks extracted from machine learning models when these continuous variables were nonnormally distributed. Student’s t test was used for normally distributed continuous variables. A comparison at p<0.05 was defined as statistically significant.

## 3 Results

Table 1 reports demographics and patient characteristics, including comorbidities, risk factors, and hospitalization details. Noncritical patients (N=28, 15 males) required no supplemental oxygen (N=15) or oxygen via a nasal cannula (N=13), whereas critical patients (N=30, 19 males) required noninvasive ventilation (NIV, N=12) or invasive ventilation via endotracheal intubation (N=18). None of the patients required extracorporeal membrane oxygenation. The number of patients with obesity and dyslipidemia was lower in the noncritical group compared to the critical group. Critical patients had a longer hospital stay than noncritical patients and were more frequently admitted to the ICU. Among the full cohort, 12 individuals died before DSO60.

**Table 1:** Demographic and clinical characteristics of COVID-19 patients. Noncritical patients required no supplemental oxygen or oxygen via a nasal cannula, whereas critical patients required noninvasive ventilation or invasive ventilation via endotracheal intubation.

Variables	Critical (N=30)	Noncritical (N=28)	Full cohort (N=58)
Age, median [IQR], years	67 [57–75]	52 [48–72]	62 [49–73]
Male sex, N (%)	19 (63)	15 (54)	34 (59)
Days since symptoms onset (DSO), median [IQR]	11 [10–13]	10 [9–13]	11 [9–13]
Symptoms, N (%)			
Cough	18 (60)	18 (64)	36 (62)^a^
Fever	21 (70)	15 (54)	36 (62)^b^
Dyspnea	24 (80)	19 (68)	43 (74)^b^
Anosmia	1 (3)	2 (7)	3 (5)^a^
Gut symptoms	7 (23)	7 (25)	14 (24)^f^
Respiratory support, N (%)			
No oxygen	0 (0)****	15 (54)	15 (26)
Nasal cannula	0 (0)****	13 (46)	13 (22)
Noninvasive	12 (40)****	0 (0)	12 (21)
Invasive	18 (60)****	0 (0)	18 (31)
Metabolic risk factors, N (%)			
Diabetes, type I	0 (0)	1 (4)	1 (2)^c^
Diabetes, type II	12 (40)	6 (21)	18 (31)^c^
Obesity	13 (43)*	4 (14)	17 (29)^e^
Overweight	10 (33)	5 (18)	15 (26)^e^
Hypertension	15 (50)	12 (43)	27 (47)^d^
Dyslipidemia	15 (50)*	5 (18)	20 (34)^d^
Chronic disease, N (%)			
Chronic liver failure	0 (0)	0 (0)	0 (0)^c^
Chronic renal failure	0 (0)	1 (4)	1 (2)^c^
Chronic heart failure	2 (7)	0 (0)	2 (3)^c^
Chronic respiratory failure	3 (10)	4 (14)	7 (12)^c^
Organ transplant	2 (7)	0 (0)	2 (3)^d^
Immunosuppression	3 (10)	2 (7)	5 (9)^c^
HIV	1 (3)	0 (0)	1 (2)
Resolve/active cancer	2 (7)	2 (7)	4 (7)^c^
Days between symptom onset and hospital admission, median [IQR]	5 [2–7]	5 [3–7]	5 [3–7]^g^
ICU admission, N (%)	21 (70)***	4 (14)	25 (43)^c^
Duration of intubation (days), median [IQR]	2 [0–20]	0 [0–0]	0 [0–5]
Duration of hospital stay (days), median [IQR]	17 [12–28]**	10 [5–16]	14 [8–22]^b^
Outcome at DSO60, N (%)			
Survivors	19 (63)**	27 (96)	46 (79)
Nonsurvivors	11 (37)**	1 (4)	12 (21)

Statistical significance between critical and noncritical groups: *P<0.05, **P<0.01, ***P<0.001, ****P<0.0001. Abbreviations: DSO, days after symptoms onset; HIV, human immunodeficiency viruses; ICU, intensive care unit; IQR, interquartile range (25th to 75th percentile). Only age, sex, and days after symptom onset data were complete for the full cohort. Sample size was incomplete for:

a Cough and anosmia (N=48).

b Fever, dyspnea, and duration of hospital stay (N=50).

c Type I diabetes, type II diabetes, chronic liver failure, chronic renal failure, chronic heart failure, chronic respiratory failure, immunosuppression, resolved/active cancer, and ICU admission (N=52).

d Hypertension, dyslipidemia, and organ transplant (N=51).

e Obesity and overweight (N=47).

f Gut symptoms (N=43).

g Days between symptom onset and hospital admission (N=49).

**Fig. 1:**
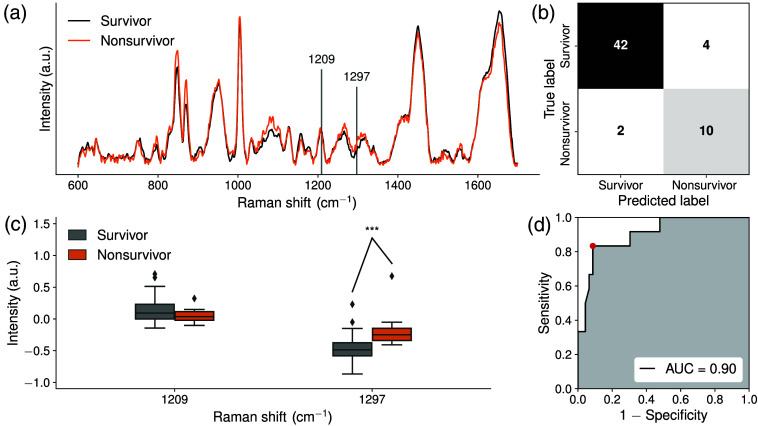
(a) Mean Raman spectra overlapped by extracted features from the model (vertical lines) for survivors (N=46, black) and nonsurvivors (N=12, orange), (b) confusion matrix, (c) box and whisker plots showing the distribution of extracted Raman peaks from the model with outliers (diamonds), and (d) area under the receiver operating characteristic curve (AUC). Significance levels are indicated as follows: ***P<0.001.

**Fig. 2:**
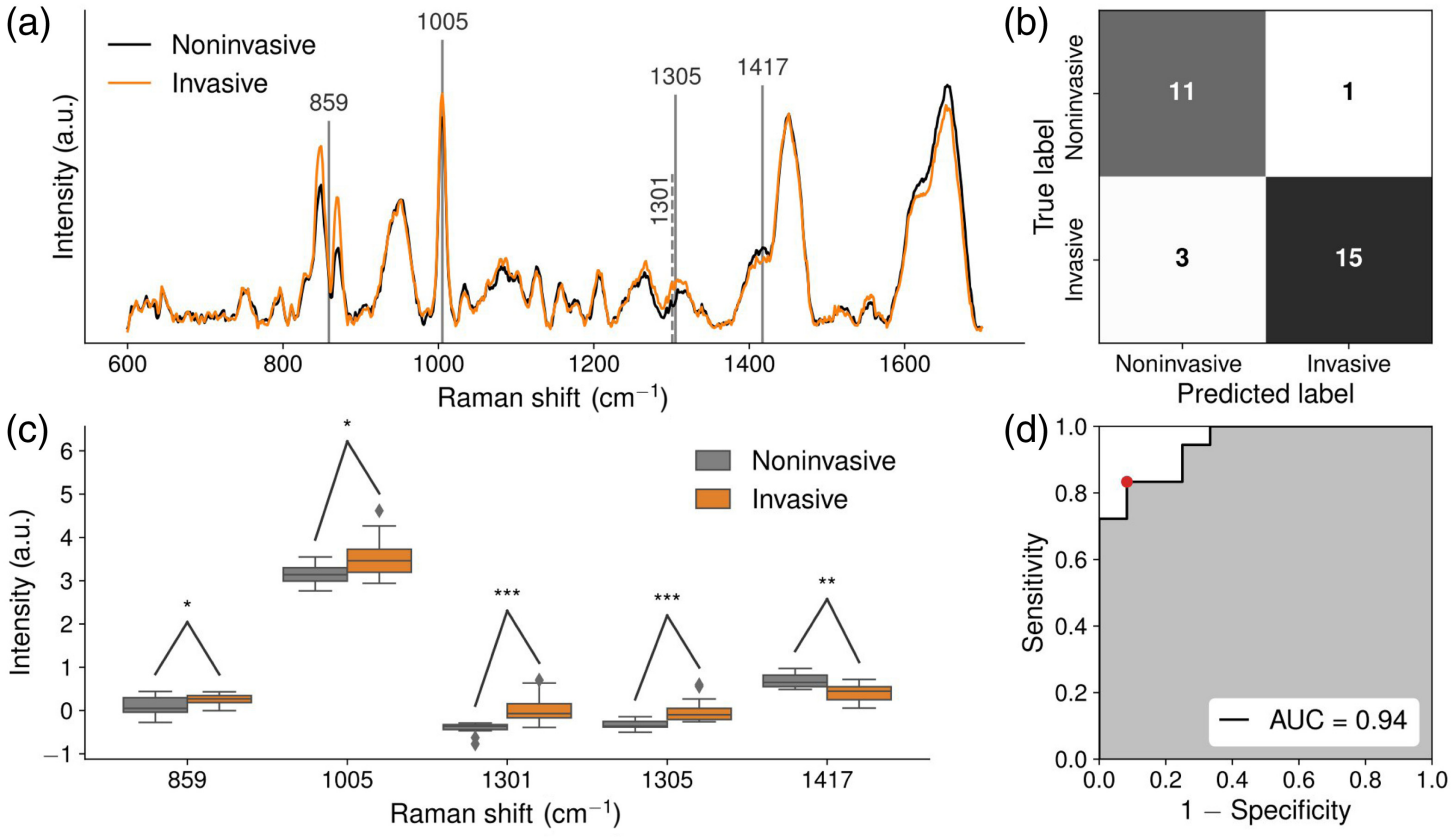
(a) Mean Raman spectra overlapped by extracted features from the model (vertical or dotted lines) for critical patients with non-invasive (N=12, black) and invasive (N=18, orange) ventilation, (b) confusion matrix, (c) box and whisker plots showing the distribution of extracted Raman peaks from the model with outliers (diamonds), and (d) area under the receiver operating characteristic curve (AUC). Significance levels are indicated as follows: *P value<0.05, **P<0.01, and ***P<0.001.

**Fig. 3:**
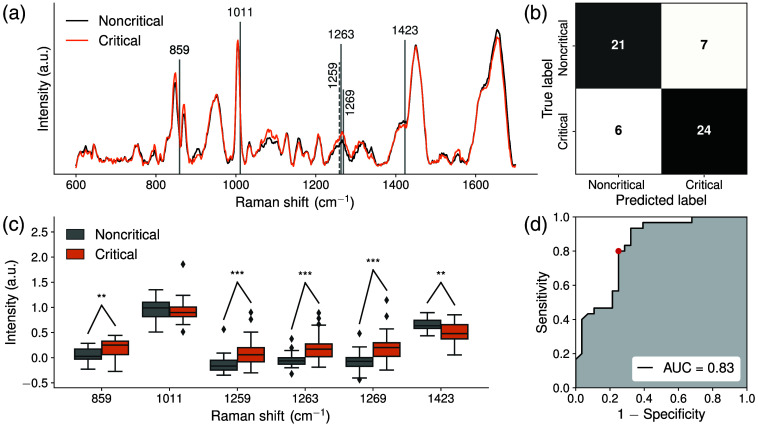
(a) Mean Raman spectra overlapped by extracted features from the model (vertical or dotted lines) for noncritical (N=28, black) and critical patients (N=30, orange), (b) confusion matrix, (c) box and whisker plots showing the distribution of extracted Raman peaks from the model with outliers (diamonds), and (d) area under the receiver operating characteristic curve (AUC). Significance levels are indicated as follows: **P<0.01 and ***P<0.001.

Three RS-ML models were created, and their performance is summarized in Table 2: survivor versus nonsurvivor (Fig. 1), critical patients with noninvasive versus invasive ventilation (Fig. 2), and noncritical versus critical patients (Fig. 3). For each RS-ML model, mean Raman spectra were plotted with key features extracted from the model highlighted with vertical dotted lines [Figs. 1(a), 2(a) and 3(a)]. Confusion matrices are shown [Figs. 1(b), 2(b) and 3(b)]. Box and whisker plots show the distribution of extracted Raman peaks from each model [Figs. 1(c), 2(c) and 3(c)] and metabolomic compounds (Figs. S1–S3 in the Supplementary Material). Receiver operating characteristic curves demonstrate the sensitivity and specificity as well as the AUC [Figs. 1(d), 2(d) and 3(d)]. For each model, Table 3 shows the biomolecular assignments of extracted Raman peaks.

**Table 2:** Accuracy, specificity, sensitivity, and area under the receiver operating characteristic curve (AUC) extracted from machine learning models.

Machine learning model	Accuracy (%)	Specificity (%)	Sensitivity (%)	AUC
Survivor (N=46) versus nonsurvivor (N=12)	90	91	83	0.90
Noninvasive (N=12) versus invasive (N=18) ventilation	87	92	83	0.94
Noncritical (N=28) versus critical (N=30) patients	78	75	80	0.83

**Table 3:** Peak center, range, and corresponding biomolecular assignments for Raman features extracted by the machine learning models.

Machine learning model	Peak center (${\mathrm{cm}}^{-1}$)	Peak range (${\mathrm{cm}}^{-1}$)	Biomolecular assignment
Survivor versus nonsurvivor	1206	1197 to 1215	Protein (tyrosine)^10^^,^^11^^,^^20^
	1301	1296 to 1306	Fatty acid, lipid (${\mathrm{CH}}_{2}$)^14^
Noninvasive versus invasive ventilation	849	835 to 863	Protein [alanine, tyrosine, leucine, lysine, and proline], glucose, and lactic acid^10^^,^^11^^,^^20^
	1005	998 to 1012	Protein [phenylalanine and tryptophan] and urea^10^^,^^11^^,^^13^^,^^20^
	1301	1296 to 1306	Fatty acid, lipid (${\mathrm{CH}}_{2}$)^14^
	1419	1416 to 1422	Lipid $\left[{\mathrm{CH}}_{2}\right]$^10^^,^^11^^,^^13^^,^^16^
Noncritical versus critical patients	849	835 to 863	Protein [alanine, tyrosine, leucine, lysine, and proline], glucose, and lactic acid^10^^,^^11^^,^^20^
	1005	998 to 1012	Protein [phenylalanine and tryptophan] and urea^10^^,^^11^^,^^13^^,^^20^
	1255	1253 to 1265	Protein [amide III and isoleucine] and lipid [=CH]^10^^–^^13^
	1266	1257 to 1275	Protein [amide III, histidine, and valine], glucose, and lipid [=CH]^10^^–^^14^^,^^20^
	1419	1416 to 1423	Lipid $\left[{\mathrm{CH}}_{2}\right]$^10^^,^^11^^,^^13^^,^^16^

### 3.1 Survivor vs. Nonsurvivor

A RS-ML model was used to discriminate between blood plasma from survivors and nonsurvivors (Fig. 1). Extracted Raman features that contributed to the ML model were the peaks at $1209\;{\mathrm{cm}}^{-1}$ (associated with proteins, in particular tyrosine)^10^^,^^11^^,^^20^ and $1297\;{\mathrm{cm}}^{-1}$ (lipids: vibrational modes related to ${\mathrm{CH}}_{2}$) as well as fatty acids.^14^ The classification model achieved an AUC of 0.90 with 83% sensitivity, 91% specificity, and 90% accuracy. The Raman signal intensity at $1209\;{\mathrm{cm}}^{-1}$ was slightly lower in nonsurvivors compared to survivors, although this comparison was not significant. Raman signal intensity at $1297\;{\mathrm{cm}}^{-1}$ in nonsurvivors was higher compared with survivors. Gold-standard metabolomics analysis showed no statistical difference in cholesterol levels (Fig. S1 in the Supplementary Material). This indicates that the difference at $1297\;{\mathrm{cm}}^{-1}$ in Raman spectra may be due to other lipids such as triglycerides or phospholipids. The concentration of urea was significantly higher in nonsurvivors compared to survivors, although the Raman peak due to urea was not a contributing factor to the ML model.

### 3.2 Critical Patients with Noninvasive vs. Invasive Ventilation

To classify blood plasma from critical patients with noninvasive ventilation and those with invasive ventilation, an RS-ML model was built (Fig. 2). Extracted Raman peaks were at $859\;{\mathrm{cm}}^{-1}$ (protein [alanine, tyrosine, leucine, lysine, and proline], glucose, and lactic acid),^10^^,^^11^^,^^20^ $1005\;{\mathrm{cm}}^{-1}$ (protein [phenylalanine and tryptophan] and urea),^10^^,^^11^^,^^13^^,^^20^ 1301 to $1305\;{\mathrm{cm}}^{-1}$ (fatty acid and lipid $\left[{\mathrm{CH}}_{2}\right]$), ^14^ and $1417\;{\mathrm{cm}}^{-1}$ (lipid $\left[{\mathrm{CH}}_{2}\right]$).^10^^,^^11^^,^^13^^,^^16^ The classification model achieved an AUC of 0.94 with 83% sensitivity, 92% specificity, and 87% accuracy. Compared to patients with NIV, the mean Raman signal in patients with invasive ventilation was higher at 859, 1005, and 1301 to $1305\;{\mathrm{cm}}^{-1}$, whereas lower at $1417\;{\mathrm{cm}}^{-1}$. There were no statistically significant differences between metabolomics measurements of cholesterol, lysine, phenylalanine, tryptophan, proline, tyrosine, and urea in patients with invasive ventilation compared with those with NIV (Fig. S2 in the Supplementary Material). However, the mean concentration of urea was higher in patients with invasive ventilation compared to those with NIV, which may partly account for the increase at $1005\;{\mathrm{cm}}^{-1}$ along with the slightly higher levels of phenylalanine. Moreover, the mean cholesterol levels were also higher in patients with invasive ventilation, which is consistent with the increases at 1301 to $1305\;{\mathrm{cm}}^{-1}$, along with other lipids. The reduction in the peak at $1417\;{\mathrm{cm}}^{-1}$ may be due to a reduction in specific types of lipids or alterations in the conformation of proteins.

### 3.3 Noncritical vs. Critical Patients

To discriminate between Raman spectra from blood plasma from patients in noncritical and critical conditions, a RS-ML model was built (Fig. 3). Raman features that contributed to the model were centered at $859\;{\mathrm{cm}}^{-1}$ (protein [alanine, tyrosine, leucine, lysine, and proline], glucose, and lactic acid),^10^^,^^11^^,^^20^ $1011\;{\mathrm{cm}}^{-1}$ (protein [phenylalanine and tryptophan] and urea),^10^^,^^11^^,^^13^^,^^20^ 1259 to 1263 to $1269\;{\mathrm{cm}}^{-1}$ (protein [amide III, isoleucine, histidine and valine], glucose, and lipid [=CH]),^10^^–^^14^^,^^20^ and $1423\;{\mathrm{cm}}^{-1}$ (lipid $\left[{\mathrm{CH}}_{2}\right]$).^10^^,^^11^^,^^13^^,^^16^ The classification model achieved an AUC of 0.83 with 80% sensitivity, 75% specificity, and 78% accuracy. When including metabolic risk factors as a covariate in addition to age and sex, AUC remained 0.83. Mean Raman intensity signal was higher at 859 and 1259 to 1263 to $1269\;{\mathrm{cm}}^{-1}$ in critical compared with noncritical patients, whereas the peak at $1423\;{\mathrm{cm}}^{-1}$ was lower. Compared with noncritical patients, critical patients showed lower concentrations of lysine, proline, and tryptophan as measured by metabolomics, whereas phenylalanine, tyrosine, urea, and cholesterol concentrations were not statistically different (Fig. S3 in the Supplementary Material).

## 4 Discussion

In this study, we presented an approach combining RS and ML modeling to classify hospitalized COVID-19 patients by disease severity and mortality. The method is rapid, portable, requires minimal sample preparation, and yields high accuracy. Raman peaks assigned to proteins, glucose, lactic acid, fatty acids, urea, and lipids were extracted by the predictive models. Area under the ROC curve ranged between 0.83 and 0.94 with sensitivities, specificities, and accuracies ranging between 80% to 83%, 75% to 92%, and 78% to 90%, respectively. Accuracy for detecting mortality, ventilation type, and severity was 90%, 87%, and 78%, respectively. These results suggest the potential of combining RS and ML modeling to stratify patients at admission, individualize care, and improve survival rates. To the best of our knowledge, this study is the first to report COVID-19 mortality prediction using RS in plasma.

Several Raman bands associated with protein were extracted, including bands linked to alanine, histidine, isoleucine, leucine, phenylalanine, proline, tryptophan, tyrosine, and valine. In the models assessing COVID-19 mortality and ventilation type, there were no significant differences in free amino acid concentrations using metabolomics. In the assessment of blood plasma from critical versus noncritical patients, there were reductions in free amino acids measured via UPLC-MS, whereas the associated Raman peaks saw a statistically significant increase. These findings strongly suggest that Raman spectroscopic changes in protein peaks are not necessarily associated with changes in free amino acid concentrations. The changes may instead be related to concentrations of particular proteins with different primary sequences. For example, the spectra of bovine serum albumin and ovalbumin both have higher peaks due to phenylalanine around $1000\;{\mathrm{cm}}^{-1}$ than that of lysozyme.^48^ Difference in Raman peaks due to proteins may also be due to protein conformational changes. For example, there are different Raman spectra for oxy- and deoxyhemoglobin, and ferritin that are bound and nonbound to iron.^49^ Changes in total protein concentration or protein binding may also affect the Raman spectra of proteins. The changes in Raman peak intensities may also be linked with concentrations of other molecules with vibrations at similar Raman shifts to the assigned amino acids.

Prior studies have shown that COVID-19 severity and mortality are indeed associated with changes in total protein concentration and the blood proteome. Hypoproteinemia (low blood protein levels) is associated with increased COVID-19 severity and mortality.^50^ Multiple proteomics studies have demonstrated changes in the serum and plasma proteomes associated with COVID-19 severity, survival, and ventilation support.^51^^–^^54^ To summarize, these results demonstrate the importance of using gold-standard techniques such as UPLC-MS to confirm molecular assignments in Raman spectra and suggest that RS may be able to detect molecular changes that cannot necessarily be detected using UPLC-MS–based metabolomics.

The Raman peak at $1005\;{\mathrm{cm}}^{-1}$ is associated with proteins and urea. This peak increases in patients with invasive compared with those on noninvasive ventilation. Changes in proteins could be responsible, as could increases in urea and lactic acid concentrations, and there is an increase in the mean urea concentration as measured by metabolomics. Urea is formed from human protein metabolism, and 90% of this waste is excreted through the kidneys.^55^ Blood urea nitrogen (BUN) is a marker of kidney damage, which in turn is a symptom of severe COVID-19 infection.^56^^,^^57^ Increased BUN levels are associated with increased COVID-19 mortality^58^ and poor prognosis in the ICU.^59^

The Raman band at 1296 to $1306\;{\mathrm{cm}}^{-1}$ is assigned primarily to lipid, including fatty acids, and corresponds to the ${\mathrm{CH}}_{2}$ twisting mode.^14^ As fundamental structural components of membranes, lipids are essential for viral replication, host lipid metabolism, and modulation of immune responses.^60^ Lipid features were extracted in all three models. Raman signal intensity at $1301\;{\mathrm{cm}}^{-1}$ was higher in nonsurvivors compared to survivors and patients with invasive compared to noninvasive ventilation. This is consistent with previous studies, which have shown that altered lipid levels are associated with COVID-19 severity, due to imbalanced lipid metabolism.^61^^,^^62^ Lipid metabolism alterations during COVID-19 infection include an increase in triglycerides and a decrease in high- and low-density lipoprotein concentrations.^63^ Nearly all of the upregulated lipids reported in ICU COVID-19 patients were triglycerides. Triglyceride levels were higher in critical patients compared with noncritical patients^63^ and in nonsurvivors compared with survivors, likely due to uncontrolled inflammation and an increased risk of mortality.^64^ Cholesterol is one of the most abundant lipid species in plasma; there was no significant difference as measured using UPLC-MS in our study, suggesting that the differences in Raman spectra are likely due to lipid features.

Predicting COVID-19 mortality and severity poses challenges, and there are very few point-of-care solutions. Blood gas measurements have been used to predict mortality, achieving specificities of 100%; however, sensitivities and AUCs remain low (between 0.6 and 0.73 for the latter).^65^ UPLC-MS is one of the gold standard methods for detecting metabolic changes. However, the equipment is extremely costly, not portable, and housed in specialised testing laboratories. Furthermore, sample analysis takes hours, and the methodology requires trained laboratory scientists and complex sample preparation. Other metabolomics techniques such as nuclear magnetic resonance spectroscopy suffer from the same limitations. In comparison to metabolomics methods, the Raman spectrometer is less costly, portable, and can be taken into clinical facilities. Sample analysis takes less than 20 s, and the methodology is label-free. The sample can be analyzed in liquid form. Machine learning models and a user-friendly interface could be integrated directly into the device for real-time point-of-care use.

Our study is a promising demonstration of how RS-ML can be used to rapidly and accurately detect disease severity and predict mortality. However, there are a few areas where future research could improve the likelihood of clinical adoption of the device. This study primarily focused on inpatients who were hospitalized following a worsening of their clinical condition, which typically occurred a few days after symptom onset. At this stage, some pathogenic events, including inflammation, plasma cytokine release, and tissue injury, may have already occurred, potentially limiting the window for certain targeted treatments. In addition, patients who were discharged early during their hospitalization were not included in this study. Complementary outpatient studies conducted at earlier stages of the disease would be valuable to identify factors that predict or are associated with the initial clinical worsening and to assess their overlap with the characteristics described in this work. The sample sizes are modest, which is due to logistic challenges and clinical research interruptions that occurred during the recruitment in the first wave of the pandemic. However, classification models reached high AUCs. The performance in discriminating between noncritical and critical patients was limited compared with the other models. This limitation may be due to the stratification of patients using respiratory support needs. It is possible that a selection of other inclusion/exclusion criteria would have increased the performance of this classification.^24^^–^^26^^,^^29^^,^^66^ However, our strategy of stratification yielded a high AUC when stratifying by noninvasive versus invasive ventilation. Repeated RS measurements were acquired at a single spot (size at the tip 170  μm, collection area ∼1  mm diameter), which may not represent the total molecular composition of the sample. However, blood plasma lacks large components such as cells, which reduce homogeneity and can sediment out, as occurs in whole blood.^67^ Prior to data acquisition, the liquid sample was vortexed to increase molecular motion, which ensured that the spectra captured molecules diffusing through the sample in different conformations and spatial distributions. It is important to note that there are key differences in the type of data obtained by RS and metabolomics, so correlation in molecular concentrations between the two methodologies remained challenging. Raman spectroscopy measures vibrational modes of molecules in both bound and free forms, whereas metabolomics tends to measure the concentrations of free molecular species. For example, the Raman band at $1005\;{\mathrm{cm}}^{-1}$ is associated with phenylalanine within proteins as well as free phenylalanine. The concentration of phenylalanine measured via metabolomics is primarily due to free phenylalanine. Furthermore, the RS peak due to phenylalanine also overlaps with the peak due to urea. Another factor to account for is that RS tends to measure vibrations associated with molecular families, e.g., the peak at $1301\;{\mathrm{cm}}^{-1}$ assigned to lipids. Meanwhile, metabolomics measures individual lipids, e.g., cholesterol. The selection of features by each model was also limited by the extraction of spectral locations that do not correspond, in some cases, to central peaks known in the literature. Although this may bring confusion for visual assessment, our approach based on spectral standardization,^15^^,^^68^ allowed identification of the most important differences between groups, which may be hidden in locations where variability is very low such as in peak positive and negative slopes or their minima. High signal-to-noise ratio during data acquisition and spectral standardization ensured the selection of the most discriminating features between groups. The complexity of this spectral interpretation could be simplified in a user-friendly graphical interface where features would be reported by simply the Raman bands they are associated with, rather than the precise Raman shift.

## 5 Conclusion

In this study, RS was combined with ML modeling to classify disease severity and mortality in COVID-19 patients. Models achieved accuracies of up to 90%, suggesting that RS-ML can help identify patients at higher risk of complications or death, allowing for more targeted and effective treatment. RS-ML is a low-cost, reagent-free methodology that can be integrated into a user-friendly, portable system, allowing efficient data acquisition and analysis within minutes from liquid blood plasma samples. These characteristics are highly desirable in the context of triage for ICU admission. Due to the versatile nature of this technique, RS-ML could be used for screening other respiratory infectious diseases, including influenza and respiratory syncytial virus. In a world where the risks of pandemics are increasing year after year,^69^ RS-ML poses an attractive solution for pathogen screening by detecting biochemical perturbations due to a virus.

## Supplementary Material

10.1117/1.JBO.31.4.046005.s01

## Data Availability

Data associated with this study are not currently available in a publicly available repository. The corresponding author will share these data upon reasonable request.
