# Supplementary material for: Combining label-free Raman spectroscopy and machine learning to identify early biomarkers of COVID-19 disease severity and mortality
Source: J Biomed Opt. 2026 Apr 15;31(4):046005. doi: 10.1117/1.JBO.31.4.046005 (PMC13082742; doi:10.1117/1.JBO.31.4.046005)
Supplement: Supplementary file 1 [file JBO_031_046005_SD001.pdf]

Combining Label-Free Raman Spectroscopy and Machine Learning to Identify Early Biomarkers of COVID-19 Disease Severity and Mortality

Maryam Heidarifard[a,b∨], Katherine Ember[c,d], Frédérick Dallaire[c,d**], Elsa Brunet-Ratnasingham[c,e], Yiheng Chen[f,g,h], Nassim Ksantini[c,d], Myriam Mahfoud[c,d], Guillaume Sheehy[c,d], Hugo Soudeyns[a,i], Philippe Jouvet[a,j], Sze Man Tse[a,j], Caroline Quach[a,k], Brent Richards,[f,g,h,l,m^], Daniel E. Kaufmann[c,e,n,o^], Frédéric Leblond[c,d#] and Mathieu Dehaes[a,b,p#]**

[∨]First author
[**]Equal contribution as co-second authors
[^]Equal contribution as co-second last senior authors
[#]Equal contribution as co-senior authors

[a]Centre de recherche Azrieli du CHU Sainte-Justine, Montreal, Quebec, Canada
[b]Institute of Biomedical Engineering, Université de Montréal, Montreal, Quebec, Canada
[c]Research Centre, CHU Montreal, Montreal, Quebec, Canada
[d]Department of Engineering Physics, Polytechnique Montréal, Montreal, Quebec, Canada
[e]Department of Microbiology, Infectiology and Immunology, Université de Montréal, Montreal, Quebec, Canada
[f]Departments of Human Genetics, Epidemiology, and Biostatistics, McGill University, Montreal, Quebec, Canada
[g]Lady Davis Institute, Jewish General Hospital, McGill University, Montreal, Quebec, Canada
[h]5 Prime Sciences, Montreal, Québec, Canada
[i]Department of Pathology and Laboratory Medicine, Pathology Advanced Translational Research Unit, Emory University School of Medicine, Atlanta, Georgia, United States
[j]Department of Pediatrics, Université de Montréal, Montreal, Quebec, Canada
[k]Department of Microbiology, Infectious Diseases and Immunology, Université de Montréal, Montreal, Quebec, Canada
[l]Department of Medicine, McGill University, Montreal, Quebec, Canada
[m]Department of Twin Research, King's College London, London, UK
[n]Division of Infectious Diseases, Lausanne University Hospital and University of Lausanne, Lausanne, Vaud, Switzerland
[o]Department of Medicine, Université de Montréal, Montreal, Quebec, Canada
[p]Department of Radiology, Radio-oncology and Nuclear Medicine, Université de Montréal, Montreal, Quebec, Canada

Appendix: Supplemental Material

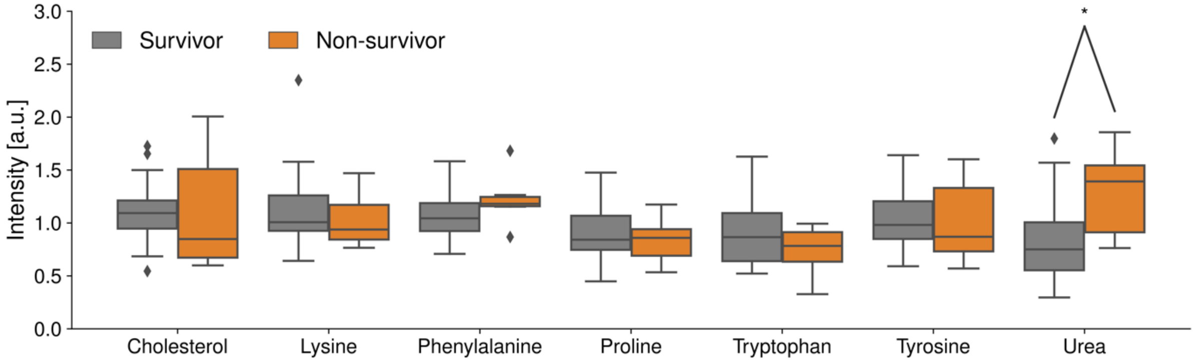

Supplementary Fig. S1. Box and whisker plots showing the distribution of relative concentration of metabolites with outliers represented by black diamonds for survivors (N=46, grey) and non-survivors (N=12, orange). Significance levels are indicated as follows: $^{*}p < 0.05$.

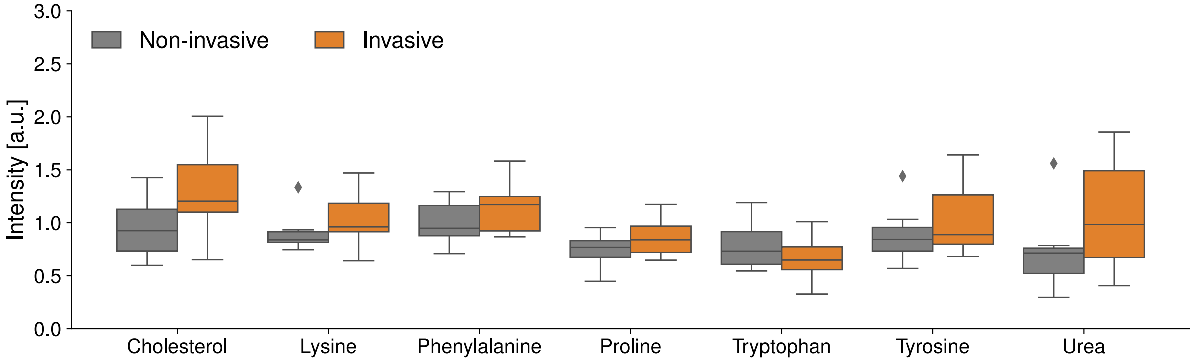

Supplementary Fig. S2. Box and whisker plots showing the distribution of relative concentration of metabolites with outliers represented by black diamonds for critical patients with non-invasive (N=12, black) and invasive (N=18, orange) ventilation.

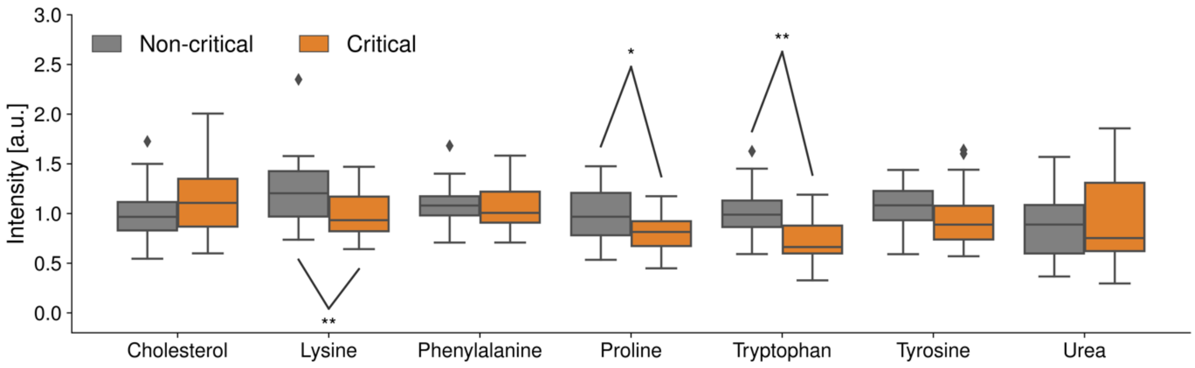

Supplementary Fig. S3. Box and whisker plots showing the distribution of relative concentration of metabolites with outliers represented by black diamonds for non-critical (N=28, grey) and critical (N=30, orange) patients. Significance levels are indicated as follows: $^{*}p < 0.05$, $^{**}p < 0.01$.
